# A proprietary herbal extract of ashwagandha root for stress and anxiety in healthy adults: a randomized, double-blind, three-arm, placebo-controlled efficacy and safety study

**DOI:** 10.25122/jml-2025-0172

**Published:** 2026-01

**Authors:** Rogene Eichler West, Anima Biswas, Rahul Rao, Himanshu Tayade, John Ademola

**Affiliations:** 1 Neuroscience, Northwest Neuro Professionals, LLC, Seattle, United States of America; 2 Obstetrics and gynaecology, Clini-Ton-Multi-Speciality Clinic, Jaipur, India; 3 Dentistry, Bharati Vidyapeeth (Deemed to be University) Dental College and Hospital, Navi Mumbai, India; 4 Pharmcology, D Y Patil University School of Medicine, Pune, India; 5 Clinical Research, San Francisco Research Institute, San Francisco , United States of America

**Keywords:** stress, anxiety, *Withania somnifera*, cortisol, perceived stress scale, proprietary herbal extract of ashwagandha, ANOVA, Analysis of variance, APB, Proprietary herbal extract of ashwagandha root, ALP, Alkaline phosphatase, ALT, Alanine transaminase, ARE, Ashwagandha root extract, AST, Aspartate transaminase, BRIEF-A, Behavior Rating Inventory of Executive Function–Adult Version, BDNF, Brain-derived neurotrophic factor, BUN, Blood urea nitrogen, CBT, Cognitive behavioral therapy, cGMP, Current good manufacturing practices, COMPASS, Computerised Mental Performance Assessment System, CTRI, Clinical Trials Registry of India, CONSORT, Consolidated Standards of Reporting Trials, ELISA, Enzyme-linked immunosorbent assay, ERA, Esteem-related affect, GAD, Generalized Anxiety Disorder, GCP, Good Clinical Practice, GSH, Glutathione peroxidase, HAM-A, Hamilton Anxiety Rating Scale, HPA, Hypothalamic–pituitary–adrenal, HPG, Hypothalamic–pituitary–gonadal, ITT, Intention-to-treat, LFT, Liver function tests, MDA, Malondialdehyde, MFS, Mental Fatigue Scale, OHQ, Oxford Happiness Questionnaire, PL, Placebo, POMS, Profile of Mood States, PP, Per-protocol, PSS-10, Perceived Stress Scale–10, PTSD, Post-traumatic stress disorder, RFT, Renal function tests, SAEs, Serious adverse events, SMD, Standardized mean difference, SD, Standard deviation, SOD, Superoxide dismutase, TEAEs, Treatment-emergent adverse events, TESAEs, Treatment-emergent serious adverse events, TMD, Total mood disturbance

## Abstract

Stress and anxiety are interconnected, sharing both behavioural and neural foundations. Ashwagandha (*Withania somnifera*) helps reduce stress and anxiety. This study aimed to evaluate the efficacy and safety of a proprietary herbal extract of ashwagandha root (APB) in adult men and women with moderate stress and anxiety. This 8-week, randomized, double-blind, three-arm, placebo-controlled trial included 141 healthy men and women aged 18–65 years with stress and anxiety. Participants were randomized to receive either 300 mg of APB twice daily (*n* = 47), ashwagandha root extract (ARE, *n* = 47), or placebo (PL, *n* = 47) for 8 weeks. The primary endpoint was a change in serum cortisol levels at week 8. Secondary endpoints were the mean changes in Perceived Stress Scale (PSS), Hamilton Anxiety Rating Scale (HAM-A), Profile of Mood States (POMS), and Oxford Happiness Questionnaire (OHQ) scores. Safety was assessed through clinical adverse events (AEs) and laboratory parameters. Between-group comparisons were analyzed using one-way ANOVA with Tukey’s post-hoc test, and within-group changes were assessed using paired *t*-tests. At 8 weeks, all groups have shown a significant reduction in serum cortisol, and improvements in PSS (*P* < 0.0001), HAM-A (*P* = 0.001), and POMS domain scores (tension, depression, and anger, *P* < 0.0001). In addition, the APB showed significant differences compared with ARE and PL for PSS, HAM-A total score, OHQ, and POMS (*P* < 0.05). The proprietary herbal extract of ashwagandha root (300mg twice daily) is considered an effective and safe intervention for reducing stress and anxiety.

## Introduction

Stress and anxiety are becoming more prevalent all around the world, leading to stress-related disorders in all age groups [1]. Stress-related disorders are becoming more common due to the demands of modern living and social and economic pressures [2]. These disorders can have an impact on both mental and physical health. Stress can be defined as a reaction to internal or external stimuli that challenge an individual’s adaptive capabilities [3]. Stress modulates health by activating the hypothalamic-pituitary-adrenal (HPA) axis, leading to increased cortisol secretion. The release of cortisol hormone triggers physiological changes to cope with stress, but chronic HPA overactivation or suppression can be harmful to both physical and mental health [4]. Anxiety is an emotional state marked by excessive worry, apprehension, or tension in response to perceived or uncertain threats, often disproportionate to the actual risk and capable of causing significant distress or functional impairment [5,6]. Individuals experiencing stress and anxiety are at a higher risk of developing both physical and psychiatric disorders, including diminished cognitive capacity, impaired memory, poor sleep quality, reduced executive function, and compromised overall psychological well-being [7]. To address these significant risks, conventional treatment options such as therapy, medication, and lifestyle modifications are commonly used. Psychotherapy, especially cognitive behavioral therapy (CBT) and, in severe cases, medications like antidepressants and benzodiazepines, can help alleviate symptoms [8]. However, these conventional treatments may have drawbacks, including withdrawal symptoms, the need for ongoing use, or the potential for misuse [9]. As concerns about these issues grow, many people are turning to alternative treatments, such as adaptogens.

Adaptogens are herbs that help the body better handle stress. These herbs help the body return to normal health when it is under a lot of stress. An adaptogen can be defined as a set of metabolic regulators that help an organism adapt to environmental changes and avoid harm [10]. Adaptogens offer numerous benefits, including helping minimise the damage stress causes, being safe and effective even when taken more than once, not causing any undesirable consequences like withdrawal symptoms, and not altering the way the body normally works. Ashwagandha is an adaptogen that has all of these properties [11,12].

Ashwagandha, scientifically known as *Withania somnifer*a and belonging to the Solanaceae family, is a renowned herb in Ayurveda, used for thousands of years as a Rasayana due to its broad spectrum of health benefits. It has been reported to reduce cortisol levels during chronic stress, rehabilitate adrenal function, and balance the sympathetic nervous system. Traditionally, ashwagandha has been used for several ailments, including fatigue, weakness, insomnia, and anxiety, all of which are accelerated by stress. Its ability to modulate oxidative stress has been demonstrated through the evaluation of biomarkers like malondialdehyde (MDA), superoxide dismutase (SOD), and glutathione peroxidase (GSH) [12-16].

Other adaptogens, such as Triphala and Trikatu, are renowned polyherbal formulations extensively utilised in Ayurveda. Triphala, derived from Sanskrit where ‘tri’ means three and ‘phala’ means fruits, is a polyherbal Ayurvedic preparation composed of the dried fruits of three plant species, referred to as the three myrobalans: *Emblica officinalis* (Amalaki), *Terminalia bellerica* (Bibhitaki), and *Terminalia chebula* (Haritaki) [17]. Triphala originated in India and was first documented in the Ayurvedic literature, Charaka Samhita. Traditional healers characterise Triphala as a potent health tonic that purifies, rejuvenates, and maintains the body’s elements [18]. In addition, studies have confirmed various potential applications of these herbs, including free radical scavenging, antioxidant activity, anti-inflammatory effects, stress-reducing effects, and adaptogenic properties [19]. Amalaki, Bibhitaki, and Haritaki have been evaluated in animal research for their protective effects against cold-induced stress, reversing stress-induced behavioural modifications and biochemical alterations, including elevated lipid peroxidation and corticosterone levels [20,21]. On the other hand, Trikatu is an herbal preparation that consists of three crude drugs: *Piper nigrum* Linn. (dried fruits of black pepper), *Piper longum* Linn. (dried fruits of long pepper), and *Zingiber officinalis* Rosc. (dried rhizomes of ginger), combined in a 1:1:1 weight ratio. Trikatu is considered a significant ‘Rasayana’ - ‘rasa’ (essence) and ‘ayana’ (way), signifying the science of prolonging life span. Trikatu has been suggested to enhance health, immunity, and longevity by supporting all body tissues [22].

There is growing interest in the development of herbal combinations, driven by the potential synergistic effects of their constituent ingredients for relieving stress and anxiety and promoting relaxation [23]. Enhancing the ability to cope with stress and anxiety represents a promising area of research. Accordingly, this study was conducted with the primary objective of evaluating the effect of a proprietary ashwagandha root extract (APB) on serum cortisol levels in healthy adults. Other objectives were to assess the effect of APB on stress, anxiety, mood, and happiness using validated scales. APB is a polyherbal formulation containing ashwagandha (*Withania somnifera* aqueous extract), Triphala, and Trikatu.

## Material and methods

### Study design

This 8-week, prospective, randomized, parallel-group, multinational, multicenter, double-blind, placebo-controlled clinical trial was conducted at two sites, one in India and one in the USA. The trial was conducted in accordance with the principles outlined in the Declaration of Helsinki (Taipei, 2016) and in accordance with Good Clinical Practice (GCP) guidelines. It also adhered to the Consolidated Standards of Reporting Trials (CONSORT) criteria for the design and reporting of randomized controlled trials.

### Study population

#### Inclusion criteria

The study included adult participants aged 18–65 years who self-reported symptoms of stress (e.g., anxiety, fatigue, or insomnia). Participants were screened and enrolled at outpatient clinical research sites. Eligible participants were required to meet the following criteria at screening: a Hamilton Anxiety Rating Scale (HAM-A) score between 14 and 30, a Perceived Stress Scale (PSS) score ≥13, and a body mass index (BMI) between 20 and 35 kg/m^2^. Participants were required to be free from any prescription or over-the-counter medications affecting stress or anxiety for at least four weeks prior to enrolment. Participants who met all the above criteria, provided consent willingly, agreed to comply with study requirements, and had no intention of initiating new treatments during the study were enrolled.

Participants were defined as otherwise healthy adults without diagnosed psychiatric disorders, who reported stress-related symptoms such as anxiety, fatigue, or insomnia. A HAM-A score of 14–30 and a PSS score ≥13 was used to identify individuals with mild to moderate anxiety and stress, rather than a clinically severe anxiety population. Individuals requiring immediate psychiatric or pharmacological intervention were excluded.

#### Exclusion criteria

Participants who were on medications affecting stress or anxiety, with conditions like depression, post-traumatic stress disorder (PTSD), Generalized Anxiety Disorder (GAD), substance abuse, or unstable diseases were excluded. Participants using dietary supplements, herbal products, or phyto-supplements known to affect stress, anxiety, mood, or neuroendocrine outcomes within four weeks prior to screening were excluded to minimize potential confounding effects. Pregnant/lactating women, those practicing meditation/relaxation for over three months, and individuals with hypersensitivity to ashwagandha or recent trial participation were also excluded. Participants with occasional analgesic or contraceptive use were not included in the study.

### Randomization and blinding

Randomization was performed using an automated random number generator (Rando version 1.2 R), with allocation sequences pre-specified for the study. Participants were randomly assigned in a 1:1:1 ratio to receive either a proprietary herbal extract of ashwagandha root extract (APB), ashwagandha root extract (ARE), or a placebo (PL). To ensure blinding, the APB, ARE, and placebo capsules were identical in appearance, shape, color, and packaging. The randomization codes were securely stored in sealed envelopes and accessed only after each participant was assigned a study identification number. The trial employed a triple-blind design, with participants, investigators, clinical staff, and the biostatistics team blinded to treatment allocation [24]. Allocation concealment was ensured using sequentially numbered, sealed, opaque envelopes.

### Study interventions

The intervention groups received capsules containing 300 mg of a standardized APB (Agaja Nutraceuticals) or 300 mg of standardized ARE. The PL group received identical capsules containing 300 mg of inert starch. Participants were instructed to take one capsule twice daily with water after breakfast and after dinner for 8 weeks and to maintain their usual dietary and lifestyle practices throughout the study period.

### Investigational products

The investigational products used in this study included a standardized APB and a standardized ARE, both used in a 1:1 ratio. These extracts are commercially sourced and manufactured under current good manufacturing practice (cGMP) conditions. Both extracts were obtained using an aqueous-based extraction method that excludes alcohol and synthetic or chemical solvents and aligns with the green chemistry principles.

APB is a proprietary multi-herbal extract containing standardized ashwagandha (*Withania somnifera*) root extract together with the classical Ayurvedic preparations Triphala and Trikatu. Triphala is composed of *Emblica officinalis* (Amalaki), *Terminalia bellerica* (Bibhitaki), and *Terminalia chebula* (Haritaki), while Trikatu includes *Piper nigrum* (black pepper), *Piper longum* (long pepper), and *Zingiber officinale* (ginger). In total, APB contains ashwagandha plus six additional botanicals. The herb-to-extract ratios were 10:1 for Triphala, 15:1 for Trikatu, and 12:1 for the standardized Ashwagandha root extract.

### Study procedures

Informed consent was obtained before commencement of the study procedures. After obtaining the consent form, baseline characteristics, including medical/surgical history and concomitant medications, were documented. All patients underwent screening to determine whether they met the inclusion or exclusion criteria. Participants were screened for brief medical history, a general physical examination, and vital signs. The safety parameters were assessed based on adverse event reporting. The study included two on-site visits for participants: one at baseline and another at the end of the study (week 8), after the first visit. A telephonic follow-up was conducted at week 4 to ensure participants’ compliance. Participant compliance with the investigational products was assessed at each study visit using pill counts and return accountability. Participants were instructed to return all unused capsules at follow-up visits, and compliance was calculated as the ratio of capsules dispensed to those returned. Standardized instructions and reminders were provided throughout the study to support adherence. At baseline, demographic data, medical and surgical history, and vital signs (blood pressure, pulse rate, body temperature, respiratory rate) were recorded. Physical examinations were performed at baseline and week 8.

### Sample size

The sample size calculation was based on the expected change in serum cortisol levels from baseline to week 8. According to Salve *et al*. [25], the reported reduction in serum cortisol at week 8 for ashwagandha dosages of 600 mg/day, 250 mg/day, and placebo were 5.26 (3.88), 2.69 (4.65), and 0.63 (4.28), respectively. Using an ANOVA model, it was estimated that a sample size of 27 participants per group would achieve 90% power to detect a difference of 0.4047 between groups at a two-sided significance level (alpha) of 0.050. Assuming 81 completed cases (27 in each arm) were required to meet the study objectives with 90% power and a 5% level of significance under a 1:1:1 allocation. Accounting for an anticipated dropout rate of 10%, the study aimed to enrol 90 participants (30 per arm), ensuring 81 completed cases per group, with 90 participants from India and 45 from the USA.

### Study outcomes

All primary and secondary outcomes were pre-specified in the study protocol. Participants were assessed at baseline, week 4, and week 8. At each time point, vital signs including pulse rate, respiratory rate, and body temperature were recorded.

#### Primary outcome

The primary efficacy endpoint was the change in serum cortisol levels from baseline to week 8. The blood samples were collected at the screening visit (baseline visit and day 1) and visit 3 (week 8).

#### Secondary outcomes

Secondary outcomes were assessed at baseline, week 4, and week 8, and included mean changes in scores from the PSS, HAM-A, Profile of Mood States (POMS), and the Oxford Happiness Questionnaire (OHQ).

##### The Perceived Stress Scale (PSS-10)

PSS-10 is a widely used instrument for assessing perceived stress, evaluating the extent to which individuals perceive their lives as unpredictable, uncontrollable, and overloaded [26]. The scale consists of 10 items that assess thoughts and feelings experienced over the preceding month. Each item is rated on a 5-point Likert scale ranging from 0 (Never) to 4 (Very Often), with four items reverse-scored to control for response bias. The total score is obtained by summing all item responses, with higher scores indicating greater perceived stress.

##### The Hamilton Anxiety Rating Scale (HAM-A)

The HAM-A tool is used to assess anxiety severity in clinical practice and research settings [27]. It consists of 14 items measuring both psychic (mental distress) and somatic (physical symptoms) anxiety. Each item is scored from 0 (not present) to 4 (severe), with total scores ranging from 0 to 56. Scores indicate severity: <17 (mild), 18–24 (mild to moderate), and 25–30 (moderate to severe).

##### Profile of Mood States (POMS, abbreviated version)

POMS is a 40-item questionnaire using a 5-point Likert scale to assess current feelings. It measures the scores across several domains: tension, anger, fatigue, depression, esteem related affect (ERA), vigor, and confusion. Scores from each domain are used to compute the Total Mood Disturbance (TMD) score (TMD = [tension score + depression score + anger score + fatigue score + confusion score] – [vigor score + ERA score]), with higher total scores indicating poorer overall mood [28].

##### Oxford Happiness Questionnaire (OHQ)

The OHQ, developed by Michael Argyle and Peter Hills, assesses happiness using 29 statements rated on a 6-point scale (1 = strongly disagree to 6 = strongly agree) [29]. Some items are reverse-scored, and the final happiness score is calculated by summing the responses and dividing the total by 29. Scores indicate happiness levels: 1–2 (not happy), 2–3 (somewhat unhappy), 3–4 (neutral), 4–5 (happy), 5–6 (very happy), with 6 suggesting possible overoptimism.

##### Hormonal and biochemical assessment

Blood samples were collected (fasting) at baseline and week 8 to evaluate serum cortisol levels and to assess liver and renal function via serum biochemical tests. Renal function was assessed based on serum creatinine and blood urea nitrogen (BUN), whereas liver function was assessed based on serum alanine transaminase (ALT), aspartate transaminase (AST), alkaline phosphatase (ALP), and total bilirubin. Samples were collected into Ethylenediaminetetraacetic acid (EDTA) and non-EDTA tubes, centrifuged, and serum aliquots were stored at -80°C for subsequent analysis. Hormone quantification in serum was performed using validated enzyme-linked immunosorbent assay (ELISA) kits.

#### Safety outcomes

Safety was evaluated by documenting all treatment-emergent adverse events (TEAEs) and treatment-emergent serious adverse events (TESAEs), whether observed by the investigators or reported by participants. These were recorded during study visits conducted in weeks 4 and 8.

### Statistical methods and data analysis

Given the sample size (>30 participants per group), parametric statistical methods were considered appropriate. Data analysis was performed using Stata 13.1 for Windows (Stata Corp, College Station, TX, USA). Continuous variables were summarized as mean ± standard deviation (SD), while categorical variables were presented as frequencies and percentages. Between-group comparisons for continuous variables were conducted using ANOVA followed by post-hoc Tukey’s test for pairwise comparisons. Categorical data were analyzed using the chi-square test. All statistical tests were two-tailed, with *P* values of 0.05 considered significant. Ninety-five percent confidence intervals (95% CIs) were calculated for key outcome measures. Analyses were conducted according to the intention-to-treat (ITT) principle, with per-protocol (PP) analyses performed for efficacy outcomes. All participants who received at least one dose of the investigational product were included in the intent-to-treat (ITT) dataset and used for safety evaluation. The PP dataset used for efficacy analyses included the participants who completed the study without major protocol deviations or violations and were not lost to follow-up.

## Results

**Figure 1:**
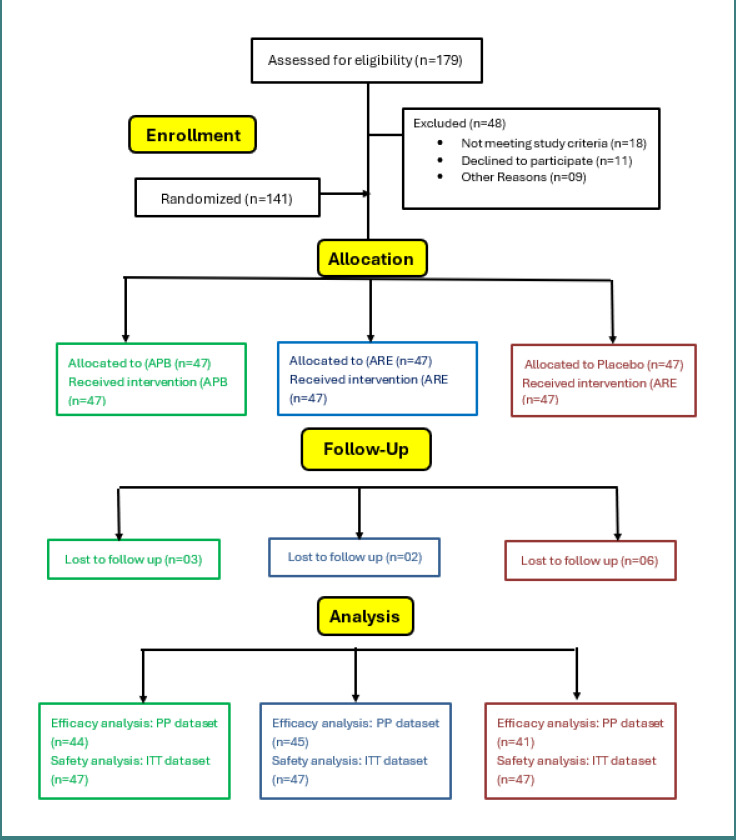
CONSORT flow diagram of participant enrollment, allocation, follow-up, and analysis

A total of 179 participants were assessed for eligibility. Of these, 48 participants were excluded, 18 did not meet the study criteria, 11 declined to participate, and 9 were excluded for other reasons. A total of 141 participants (India = 90, US = 51) were enrolled and randomized into three groups: APB (*n* = 47), ARE (*n* = 47), and PL (*n* = 47). Lost to follow-up were 3 in the APB group, 2 in the ARE group, and 6 in the placebo group. The efficacy analysis (PP) dataset consists of 130 participants: APB (*n* = 44), ARE (*n* = 45), and PL (*n* = 41). The safety analysis (intention-to-treat) dataset consists of 47 participants per group. Thus, this method provided a detailed evaluation of both efficacy and safety outcomes across the study. A CONSORT flow diagram illustrating the participant disposition is shown in Figure 1.

### Demographic profile of participants and baseline data in the ITT dataset

Demographic and baseline profiles were similar (*P* > 0.05) across the three groups for all randomized participants (*n* = 141) included in the ITT analysis (Table 1). Gender distribution was similar (*P* = 0.278) across the three groups, with more women (87.20% in APB, 93.60% in ARE, and 95.70% in PL) than men.

**Table 1:** Baseline demographic and clinical characteristics of participants in the ITT dataset (*n* = 141)

	APB (*n* = 47) Mean (SD)	ARE (*n* = 47) Mean (SD)	PL (*n* = 47) Mean (SD)	ANOVA *P*
**Demography**				
Age (yrs)	42.13 (11.09)	42.43 (10.72)	42.04 (12.00)	0.985
BMI (kg/sq.m^2^)	24.59 (2.90)	24.28 (2.35)	25.03 (3.91)	0.506
**Perceived Stress Scale (PSS)**				
• PSS total score	30.89 (6.62)	30.34 (7.38)	30.15 (6.76)	0.864
**Hamilton Anxiety Rating Scale (HAM-A)**				
• HAM-A total score	38.11 (12.46)	37.85 (12.33)	37.91 (12.28)	0.995
**Profile of Mood States (POMS)**				
• Tension	17.26 (4.57)	16.81 (4.85)	16.57 (4.89)	0.781
• Anger	17.53 (5.11)	16.17 (5.00)	16.43 (4.66)	0.365
• Depression	20.81 (5.47)	19.51 (5.49)	18.98 (4.91)	0.230
• Fatigue	14.19 (4.97)	13.47 (5.39)	13.51 (5.53)	0.760
• Confusion	13.85 (4.83)	13.38 (5.03)	13.40 (5.03)	0.875
• ERA	17.43 (5.11)	16.91 (5.22)	16.62 (5.36)	0.751
• Vigour	14.51 (4.44)	13.51 (5.09)	13.17 (4.63)	0.363
• Total Mood Disturbance (TMD)	151.70 (15.11)	148.91 (15.17)	149.11 (15.10)	0.609
**Oxford Happiness Questionnaire (OHQ)**				
• OHQ total score	2.24 (0.62)	2.26 (0.53)	2.28 (0.58)	0.955
**Hormonal levels**				
• Cortisol levels (mcg/dL)	16.55 (6.04)	16.45 (7.20)	16.95 (6.41)	0.927
**Liver function tests (LFTs)**				
• Total Bilirubin (mg/dL)	0.79 (0.08)	0.77 (0.08)	0.79 (0.10)	0.698
• Direct Bilirubin (mg/dL)	0.43 (0.08)	0.41 (0.09)	0.42 (0.09)	0.467
• Indirect Bilirubin (mg/dL)	0.35 (0.03)	0.37 (0.05)	0.36 (0.05)	0.530
• ALP (U/L)	115.33 (7.11)	115.00 (7.44)	115.01 (7.09)	0.980
• AST (U/L)	34.55 (2.72)	34.89 (2.62)	34.66 (2.74)	0.887
• ALT (U/L)	42.12 (3.76)	42.89 (2.84)	42.28 (4.65)	0.710
**Renal function tests (RFTs)**				
• Sr. Creatinine (mg/dL)	0.74 (0.37)	0.82 (0.30)	0.73 (0.37)	0.602
• BUN (mg/dL)	11.30 (3.53)	11.56 (4.39)	12.10 (4.70)	0.759

* One-way analysis of variance (ANOVA) pairwise comparisons. APB, Ashwagandha proprietary herbal extract; ARE, Ashwagandha root extract; ITT, intent-to-treat; PL, placebo; Age (yrs.): Age in years; BMI (kg/m^2^), Body Mass Index, measured in kilograms per square meter; SBP (mmHg): Systolic Blood Pressure in millimeters of mercury; DBP (mmHg), Diastolic Blood Pressure in millimeters of mercury; Pulse Rate (per min), Number of heart beats per minute; Body Temperature (°F), Body temperature in degrees Fahrenheit; Respiratory Rate (per min), Number of breaths per minute; PSS Total Score, Total score on the Perceived Stress Scale; HAM-A Total Score, Total score on the Hamilton Anxiety Rating Scale; ERA, Emotional Role Adaptability from POMS; TMD, Total Mood Disturbance, sum of POMS subdomains; OHQ Total Score, Total score on the Oxford Happiness Questionnaire; ALP (U/L), Alkaline Phosphatase in units per liter; AST (U/L), Aspartate Aminotransferase in units per liter; ALT (U/L), Alanine Aminotransferase in units per liter; Sr. Creatinine (mg/dL), Serum Creatinine in milligrams per deciliter; BUN (mg/dL), Blood Urea Nitrogen in milligrams per deciliter.

#### Primary outcome

##### Change in serum cortisol levels

**Figure 2:**
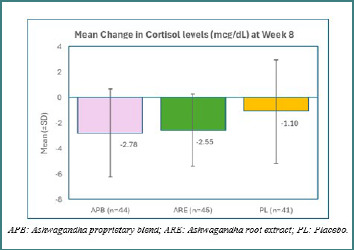
Mean scores of cortisol levels (mcg/dL) in the PP dataset (*n* = 130)

The mean baseline change in serum cortisol levels showed no significant differences across the three groups (APB, ARE, and PL), as confirmed by a one-way ANOVA. After 8 weeks, participants in the APB and ARE groups demonstrated greater reductions in serum cortisol than those in the PL group, although these differences were not statistically significant. The mean (SD) reduction in serum cortisol was −2.78 (3.43) μg/dL for APB, −2.55 (2.84) μg/dL for ARE, and −1.10 (4.08) μg/dL for PL (Table 2 and Figure 2).

**Table 2:** Cortisol levels, PSS score, and HAM-A score at baseline and during the study period in the PP dataset (*n* = 130)

	APB (*n* = 44) Mean (SD)	ARE (*n* = 45) Mean (SD)	PL (*n* = 41) Mean (SD)	ANOVA (Between Group) *P*	APB Vs. ARE *P*	ARE Vs. Placebo *P*	Placebo Vs. APB *P*
**Hormonal levels**							
**Cortisol levels (mcg/dL)**							
Baseline	16.92 (6.07)	16.85 (7.09)	17.78 (6.00)	0.758	0.999	0.780	0.808
Change at 8 weeks	-2.78 (3.43)	-2.55 (2.84)	-1.10 (4.08)	0.059	0.949	0.133	0.071
**PSS total score**							
Baseline	31.30 (6.65)	30.80 (7.15)	31.17 (6.55)	0.938	0.937	0.965	0.996
Change at 4 weeks	-5.95 (5.08)	-3.78 (5.77)	-0.98 (5.62)	<0.0001*	0.152	0.051	<0.0001*
Change at 8 weeks	-11.75 (8.52)	-8.20 (6.59)	-1.29 (5.57)	<0.0001*	0.049	<0.0001*	<0.0001*
**HAM-A total score**							
Baseline	39.16 (12.11)	38.56 (12.11)	39.85 (11.89)	0.883	0.970	0.872	0.962
Change at 4 weeks	-12.30 (17.48)	-5.18 (5.39)	-0.83 (6.34)	<0.0001*	0.009*	0.176	<0.0001*
Change at 8 weeks	-17.48 (10.19)	-8.22 (13.50)	-1.59 (8.78)	0.001*	<0.0001*	0.017	<0.0001*

Statistical significance is indicated by an asterisk (*P* < 0.05). ANOVA with Tukey’s post-hoc pairwise comparisons. APB, Ashwagandha proprietary herbal extract; ARE, Ashwagandha root extract; PL, placebo; PSS Total Score, Total score on the Perceived Stress Scale; HAM-A Total Score, Total score on the Hamilton Anxiety Rating Scale.

### Secondary outcomes

#### PSS-10 and HAM-A

**Figure 3:**
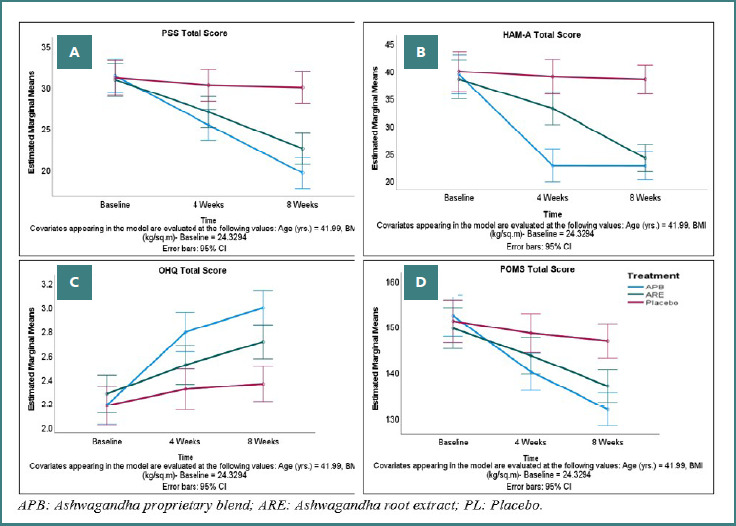
Mean scores of PSS, HAM-A, OHQ, and POMS assessment in the PP dataset (*n* = 130). A, **PSS total score;** B, **HAM-A total score;** C, **OHQ total score;** D, **POMS total score**

At baseline, there were no significant differences in PSS or HAM-A scores among the three groups (APB, ARE, and placebo), as calculated by ANOVA. After 8 weeks, the APB group showed significant improvements as compared to both ARE and PL groups, with mean reductions of -11.75 (SD 8.52) for PSS and -17.48 (SD 10.19) for HAM-A. These changes were significant, with *P* values <0.0001 for comparisons between APB and placebo, and <0.05 for certain other comparisons. Improvements in the PL group were minimal, indicating the superior effectiveness of APB and ARE in reducing stress and anxiety (Table 2 and Figure 3A-D).

#### POMS

**Figure 4:**
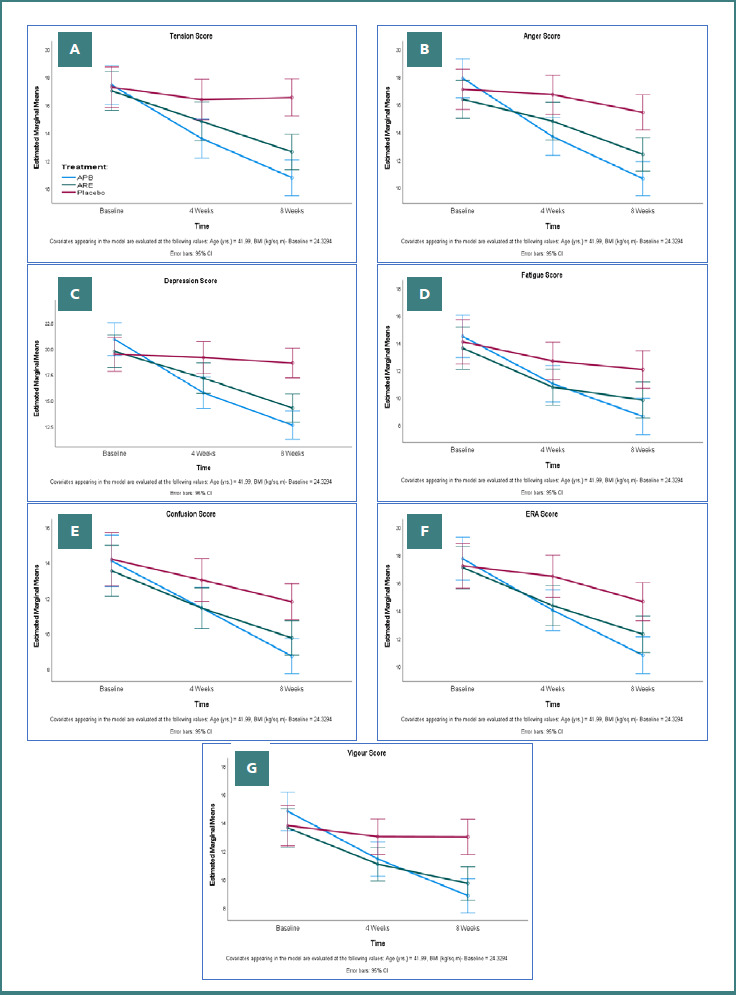
POMS domain scores assessment in the PP dataset (*n* = 130). A, Tension score; B, Anger score; C, Depression score; D, Fatigue score; E, Confusion score; F, ERA score; G, Vigor score

At baseline, the three groups (APB, ARE, and PL) showed no significant differences in the POMS domains, including tension, anger, depression, fatigue, confusion, ERA, and vigour. However, at both 4 weeks and 8 weeks, the APB group demonstrated significant reductions in these domains compared to ARE and placebo. For example, at 8 weeks, tension decreased by -6.64 (SD 4.73) in APB, compared to -4.29 (SD 3.88) in ARE and -0.80 (SD 3.74) in PL (*P* < 0.0001). Similarly, the total mood disturbance (TMD) score decreased by -20.48 (SD 14.37) in APB, compared to -12.51 (SD 11.18) in ARE and -4.46 (SD 8.88) in PL (*P* < 0.0001). These findings indicate that APB consistently and significantly improved mood-related parameters more effectively than both ARE and PL (Table 3 and Figure 4A-G).

**Table 3:** OHQ and POMS score at baseline and in the PP dataset (*n* = 130)

	APB (*n* = 44) Mean (SD)	ARE (*n* = 45) Mean (SD)	PL (*n* = 41) Mean (SD)	ANOVA (Between Group) *P*	APB Vs. ARE *P*	ARE Vs. Placebo *P*	Placebo Vs. APB *P*
**OHQ total score**							
Baseline	2.20 (0.61)	2.25 (0.54)	2.19 (0.57)	0.868	0.892	0.889	1.000
Change at 4 Weeks	0.61 (0.46)	0.24 (0.23)	0.14 (0.35)	<0.0001*	<0.0001*	0.362	<0.0001*
Change at 8 Weeks	0.81 (0.30)	0.43 (0.33)	0.18 (0.23)	<0.0001*	<0.0001*	<0.0001*	<0.0001*
**POMS domain**							
**Tension**							
Baseline	17.41 (4.64)	16.98 (4.77)	17.34 (4.74)	0.898	0.903	0.932	0.998
Change at 4 Weeks	-3.84 (3.88)	-2.13 (3.23)	-0.93 (3.46)	0.001*	0.062	0.257	0.001*
Change at 8 Weeks	-6.64 (4.73)	-4.29 (3.88)	-0.80 (3.74)	<0.0001*	0.023	<0.0001*	<0.0001*
**Anger**							
Baseline	17.86 (5.00)	16.31 (4.95)	17.20 (4.36)	0.312	0.281	0.670	0.797
Change at 4 Weeks	-4.20 (3.87)	-1.53 (3.23)	-0.44 (2.61)	<0.0001*	0.001	0.276	<0.0001
Change at 8 Weeks	-7.25 (4.94)	-3.91 (3.57)	-1.73 (3.38)	<0.0001*	<0.0001*	0.036	<0.0001*
**Depression**							
Baseline	20.91 (5.63)	19.73 (5.33)	19.51 (5.00)	0.426	0.553	0.980	0.451
Change at 4 Weeks	-5.18 (4.25)	-2.53 (3.42)	-0.37 (3.97)	<0.0001*	0.005	0.029	<0.0001*
Change at 8 Weeks	-8.32 (5.63)	-5.40 (4.69)	-0.90 (4.98)	<0.0001*	0.022	<0.0001*	<0.0001*
**Fatigue**							
Baseline	14.43 (4.98)	13.62 (5.35)	14.12 (5.54)	0.767	0.751	0.900	0.961
Change at 4 Weeks	-3.43 (3.58)	-2.82 (2.83)	-1.44 (2.50)	0.009	0.607	0.089	0.008
Change at 8 Weeks	-5.84 (4.14)	-3.73 (3.41)	-2.07 (3.45)	<0.0001*	0.022	0.097	<0.0001*
**Confusion**							
Baseline	14.07 (4.85)	13.51 (5.02)	14.24 (4.80)	0.766	0.853	0.768	0.985
Change at 4 Weeks	-2.61 (3.25)	-2.11 (2.66)	-1.17 (2.10)	0.050	0.660	0.249	0.042
Change at 8 Weeks	-5.32 (4.34)	-3.80 (3.36)	-2.39 (3.33)	0.002	0.135	0.188	0.001
**ERA**							
Baseline	17.70 (5.10)	17.04 (5.24)	17.29 (5.24)	0.833	0.820	0.973	0.929
Change at 4 Weeks	-3.70 (3.61)	-2.67 (2.81)	-0.80 (2.52)	<0.0001*	0.242	0.014	<0.0001*
Change at 8 Weeks	-6.93 (4.94)	-4.73 (4.13)	-2.61 (4.26)	<0.0001*	0.056	0.074	<0.0001*
**Vigour**							
Baseline	14.77 (4.39)	13.62 (5.11)	13.85 (4.46)	0.476	0.479	0.971	0.637
Change at 4 Weeks	-3.34 (3.50)	-2.56 (2.79)	-0.80 (2.43)	<0.0001*	0.423	0.019	<0.0001*
Change at 8 Weeks	-5.95 (4.27)	-3.89 (3.35)	-0.83 (4.53)	<0.0001*	0.047	0.002	<0.0001*
**POMS**							
Baseline	152.20 (15.40)	149.49 (14.76)	151.27 (14.87)	0.687	0.671	0.847	0.956
Change at 4 Weeks	-12.23 (11.62)	-5.91 (8.51)	-2.73 (8.87)	<0.0001*	0.008	0.292	<0.0001*
Change at 8 Weeks	-20.48 (14.37)	-12.51 (11.18)	-4.46 (8.88)	<0.0001*	0.005	0.005	<0.0001*

Statistical significance is indicated by an asterisk (*P* < 0.05). ANOVA with Tukey’s post-hoc pairwise comparisons; APB: Ashwagandha proprietary herbal extract; ARE, Ashwagandha root extract; PL, placebo; TMD, Total Mood Disturbance; ERA, Esteem-related affect; POMS, Profile of Mood States.

#### OHQ

At baseline, there were no significant differences in OHQ scores across APB, ARE, and PL groups. After 8 weeks, changes in OHQ scores showed greater improvement in the APB group (mean change 0.81, SD 0.30) than in the ARE (0.43, SD 0.33) and placebo (0.18, SD 0.23) groups, with a significant *P* value (<0.0001) (Table 3, Figure 3).

### Safety outcomes

After 8 weeks, small numerical reductions in total bilirubin, direct bilirubin, and indirect bilirubin were observed in the APB group (−0.07 ± 0.13 mg/dL, −0.08 ± 0.12 mg/dL, and −0.16 ± 0.05 mg/dL, respectively), which reached statistical significance. However, all liver function parameters were within normal clinical limits at baseline across groups, and the magnitude of change remained within the normal physiological range. These findings therefore reflect statistical variation rather than clinically meaningful effects, and indicate stable hepatic safety. Renal function tests (RFTs) showed only minor changes across groups, with no statistically significant differences. These results indicate that 8-week APB therapy has no detrimental effects on liver or renal function (Table 4). No serious adverse events (SAEs) were reported during the study.

**Table 4:** Safety / Laboratory parameters score at baseline and week 8 in the PP dataset (*n* = 130)

	APB (*n* = 44) Mean (SD)	ARE (*n* = 45) Mean (SD)	PL (*n* = 41) Mean (SD)	ANOVA (Between Group) *P*	APB Vs. ARE *P*	ARE Vs. Placebo *P*	Placebo Vs. APB *P*
**Liver function tests (LFTs)**							
**Total Bilirubin (mg/dL)**							
• Baseline	0.79 (0.08)	0.77 (0.08)	0.79 (0.10)	0.698	0.741	0.745	1.000
• Change at 8 Weeks	-0.07 (0.13)	0.13 (0.14)	0.12 (0.13)	<0.0001*	<0.0001*	0.974	<0.0001*
**Direct Bilirubin (mg/dL)**							
• Baseline	0.43 (0.08)	0.41 (0.09)	0.42 (0.09)	0.467	0.442	0.721	0.893
• Change at 8 Weeks	-0.08 (0.12)	0.01 (0.15)	0.03 (0.12)	0.005	0.025	0.898	0.007
**Indirect Bilirubin (mg/dL)**							
• Baseline	0.36 (0.03)	0.37 (0.05)	0.36 (0.05)	0.530	0.562	0.994	0.626
• Change at 8 Weeks	-0.16 (0.05)	-0.11 (0.07)	-0.10 (0.06)	0.001*	0.005	0.887	0.001
**ALP (U/L)**							
• Baseline	115.33 (7.11)	115.00 (7.44)	115.01 (7.09)	0.980	0.983	1.000	0.984
• Change at 8 Weeks	0.40 (1.19)	0.61 (1.90)	0.17 (0.95)	0.486	0.831	0.453	0.806
**AST (U/L)**							
• Baseline	34.55 (2.72)	34.89 (2.62)	34.66 (2.74)	0.887	0.881	0.942	0.988
• Change at 8 Weeks	0.78 (3.61)	-1.86 (8.40)	-0.03 (0.18)	0.146	0.135	0.377	0.823
**ALT (U/L)**							
• Baseline	42.12 (3.76)	42.89 (2.84)	42.28 (4.65)	0.710	0.714	0.806	0.987
• Change at 8 Weeks	-0.60 (2.75)	-0.66 (2.86)	-0.13 (0.73)	0.635	0.995	0.661	0.719
**Renal function tests (RFTs)**							
**Creatinine (mg/dL)**							
• Baseline	0.74 (0.37)	0.82 (0.30)	0.73 (0.37)	0.602	0.690	0.630	0.995
• Change at 8 Weeks	-0.14 (0.22)	-0.17 (0.23)	-0.05 (0.42)	0.348	0.920	0.337	0.563
**BUN (mg/dL)**							
• Baseline	11.30 (3.53)	11.56 (4.39)	12.10 (4.70)	0.759	0.970	0.875	0.747
• Change at 8 Weeks	0.92 (4.68)	1.84 (4.95)	0.45 (6.21)	0.591	0.782	0.572	0.937

Statistical significance is indicated by an asterisk (*P* < 0.05). ANOVA with Tukey’s post-hoc pairwise comparisons. APB, Ashwagandha proprietary herbal extract; ARE, Ashwagandha root extract; PL, placebo; OHQ Total Score, Total score on the Oxford Happiness Questionnaire; ALP (U/L), Alkaline Phosphatase in units per liter; AST (U/L), Aspartate Aminotransferase in units per liter; ALT (U/L), Alanine Aminotransferase in units per liter; Sr. Creatinine (mg/dL), Serum Creatinine in milligrams per deciliter; BUN (mg/dL), Blood Urea Nitrogen in milligrams per deciliter.

## Discussion

Stress can be a risk factor for physical and mental health; it impairs cognition, memory, sleep, and overall well-being, which contributes to somatic problems [1]. Many studies have previously demonstrated the benefits of ashwagandha root extract for the management of anxiety and stress. A study by Chandrasekhar *et al*. evaluated the efficacy of ashwagandha root extract in healthy adults with stress and anxiety, and the results demonstrated that ashwagandha root extract significantly reduced (*P* < 0.0001) all stress-assessment scales compared with the placebo group [12]. Another study by Salve *et al*. examined ARE in stressed healthy adults and found that ARE significantly improved (*P <* 0.001) in alleviating stress. The results of this study demonstrated a significant decrease in cortisol levels in the ARE group when compared with placebo [25]. Ashwagandha mainly modulates the hypothalamic–pituitary–gonadal (HPG) axis via non-oxidative mechanisms and exerts its anti-stress effects by influencing the HPA axis [30]. A systematic review and meta-analysis of 12 randomized clinical trials (1,002 participants, aged 25-48) evaluated the effects of ashwagandha on anxiety and stress. The results demonstrated significant reductions in anxiety (SMD: −1.55; 95% CI, −2.37, −0.74) and stress (SMD: −1.75; 95% CI, −2.29, −1.22) compared to placebo. The meta-analyses further observed that a dose of 300–600 mg per day produced optimal effects on stress, while doses up to 12,000 mg per day were beneficial for anxiety [31]. In our study, we used a 600 mg dose of ashwagandha, along with Trikatu and Triphala, and observed similar benefits for stress reduction.

Triphala, an integral component of the proprietary herbal blend used in this study, contains bioactive compounds, including polyphenols and other phytochemicals, which have been extensively studied in preclinical models. Although its effects on stress-related cognitive and mood outcomes in humans are less extensively characterized, experimental and clinical evidence suggest that Triphala exhibits antistress and adaptogenic properties, largely attributed to its antioxidant, free-radical-scavenging, anti-inflammatory, and immunomodulatory activities [32,33]. Triphala has demonstrated a favorable safety profile across *in vitro, in vivo*, and human studies, with no cytotoxicity observed in cellular assays and no adverse findings in acute, subacute, or chronic toxicity studies. Human trials further confirm tolerability at doses up to 2,500 mg/day. Mechanistically, Triphala has been shown to suppress pro-inflammatory cytokines, including tumor necrosis factor-alpha (TNF-α), interleukin-1 beta (IL-1β), and interleukin-6 (IL-6), inhibit nuclear factor kappa B (NF-κB) signaling, and modulate immune responses, thereby supporting its therapeutic relevance in stress- and inflammation-associated conditions [34–37].

Another ingredient of APB, Trikatu, is traditionally used to treat anorexia and has been reported to improve anorexia in rat studies [22]. Preclinical safety studies indicate that Trikatu is well tolerated, with no significant effects on mortality, body weight, organ weights, hematological indices, or biochemical parameters, including serum and lipid profiles, even at doses up to 2,000 mg/kg in acute and subacute toxicity studies [38]. The combination of ashwagandha with Triphala and Trikatu may enhance therapeutic efficacy through complementary mechanisms, including improved bioavailability, antioxidant signaling, and neuromodulatory effects. Emerging evidence suggests that such combinations may alleviate fatigue, mood disturbances, and sleep impairment by modulating oxidative stress pathways, serotonergic (5-hydroxytryptamine [5-HT]) signaling, and brain-derived neurotrophic factor (BDNF)-mediated neuronal and synaptic plasticity [39–41].

This study evaluated the efficacy and safety of APB and ARE in alleviating stress, anxiety, and mood states, while also confirming its tolerability. The study participants were randomized into the three groups (APB, ARE, and PL) with similar baseline demographics and clinical parameters. The primary outcome showed a statistically insignificant reduction in serum cortisol levels in the APB group at 8 weeks, suggesting a trend toward better stress-modulating properties of APB compared with the ARE and PL groups. However, these results did not achieve statistical significance, and further studies are required to achieve adequate power for this analysis.

The secondary outcomes corroborated the effectiveness of APB, showing significant improvements in PSS and HAM-A scores (*P* < 0.0001). Furthermore, OHQ showed significant improvements in the APB group, suggesting its broader benefits. These findings are consistent with previously published evidence. A review of five randomized controlled trials involving 254 participants reported that ashwagandha significantly reduced anxiety (HAM-A scores: mean difference = −5.96) and enhanced sleep metrics [40]. Similarly, a three-arm, eight-week study by Salve *et al*. evaluated ARE for stress-relief effects in participants with PSS scores >20. The study demonstrated significant reductions in stress with ashwagandha at doses of 250 mg/day (*P* < 0.05) and 600 mg/day (*P* < 0.001), along with significant decreases in cortisol levels (*P* < 0.05 and *P* < 0.0001, respectively) compared with placebo. Improvements in sleep quality were also reported [25]. This supports the efficacy of ashwagandha, which is present in both the APB and ARE groups.

In the present study, the POMS scale further demonstrated the efficacy of APB, with significant reductions in tension, depression, and anger (*P* < 0.0001). These findings are comparable to those reported by Kale *et al*. [41], who evaluated the effects of ashwagandha using the POMS scale. In that study, ARE showed significant improvements (*P* < 0.05) in episodic memory, working memory, attention accuracy (COMPASS), and mood (POMS-A). Improvements were also observed in Mental Fatigue Scale (MFS) and Behavior Rating Inventory of Executive Function–Adult Version (BRIEF-A) scores, indicating increased vigor and enhanced cognitive function. Together, these findings highlight the broader neurocognitive and mood-related benefits of ashwagandha.

No serious adverse events were reported, and the intervention was well tolerated across all groups. These observations suggest that APB is a safe and effective intervention for stress- and mood-related symptoms, with benefits comparable to those observed in the ARE group.

Ashwagandha, a well-recognized herb in Ayurvedic medicine, is known for its adaptogenic properties. Modern clinical research supports its efficacy for stress management, anxiety reduction, and overall well-being.

### Strengths

The current study employed a randomized controlled trial design, ensuring balanced group allocation as well as minimizing bias. Both the ITT and PP datasets were analyzed, providing a detailed assessment of safety and efficacy. The objective outcomes included cortisol levels and liver (and kidney) function, as well as validated psychological scales (PSS, HAM-A, POMS), which enhanced the validity of the findings. The lack of serious adverse events and a favorable safety profile make this intervention a viable option compared to standard treatments. Research across multiple sites in India and the USA also added further diversity to the participant group.

### Limitations

This study has several limitations. Although the sample size was adequate for analysis, it limits generalizability to larger and more diverse populations, and the 8-week intervention period may be insufficient to evaluate long-term outcomes. The exclusion of individuals with diagnosed psychiatric disorders restricts extrapolation of findings to moderate or severe anxiety, suggesting that observed benefits are most applicable to mildly anxious or stress-affected healthy adults. Using several self-reported questionnaires, though validated, may have introduced subjective bias and caused participant fatigue or neutral (middle-range) responses. Furthermore, concomitant use with psychotropic medications was not evaluated, and thus, the safety and efficacy of these herbal products as adjunct therapies remain unknown. Future studies with longer durations, larger and more diverse populations, streamlined assessment tools, and evaluation of herb–drug interactions are needed.

## Conclusion

The present study demonstrates that both ashwagandha root extract (ARE) and the proprietary ashwagandha herbal extract (APB) could be effective and safe for managing stress and anxiety in adults. Compared to placebo, ashwagandha root extract alone produced significant improvements in stress and anxiety measures, including PSS, HAM-A, POMS domains, and happiness scores (OHQ), along with a reduction in serum cortisol levels. Importantly, the proprietary herbal extract (APB) yielded even greater improvements across these parameters, suggesting a synergistic enhancement of efficacy. Both extracts were well tolerated, with no serious adverse events reported, supporting their safety. Overall, these findings suggest that APB is a reliable adaptogen that provides improved support for mental and physical well-being.

## Data Availability

The data supporting the findings of this study are available from the corresponding author upon reasonable request.

## Personal thanks

The authors thank Agaja Nutraceuticals, for supplying the investigational products used in this study.

## References

[1] Varma P, Junge M, Meaklim H, Jackson ML (2021). Younger people are more vulnerable to stress, anxiety and depression during COVID-19 pandemic: a global cross-sectional survey. Prog Neuropsychopharmacol Biol Psychiatry.

[2] Halbreich U (2021). Stress-related physical and mental disorders: a new paradigm. BJPsych Adv.

[3] Domes G, Frings C (2020). Stress and cognition in humans: current findings and open questions in experimental psychology. Exp Psychol.

[4] Van Dalfsen JH, Markus CR (2018). The influence of sleep on human hypothalamic-pituitary-adrenal (HPA) axis reactivity: a systematic review. Sleep Med Rev.

[5] Takagi Y, Sakai Y, Abe Y, Nishida S, Harrison BJ, Martínez-Zalacaín I (2018). A common brain network among state, trait, and pathological anxiety from whole-brain functional connectivity. Neuroimage.

[6] Goes TC, Almeida Souza TH, Marchioro M, Teixeira-Silva F (2018). Excitotoxic lesion of the medial prefrontal cortex in Wistar rats: effects on trait and state anxiety. Brain Res Bull.

[7] Nuralieva N, Chang M, Huang L, Ts S (2024). Neurocognitive remediation therapy: a promising approach to enhance cognition in community living pilots with depression and anxiety. Psychol Res Behav Manag.

[8] Ibrahim HK, Ghumid MJ, Al-Sadiq OK (2024). Medicines used to treat post-traumatic stress disorder (PTSD). Libyan J Med Appl Sci.

[9] Maviglia M, Cooeyate NJ (2024). Implementing psychiatric drug withdrawal practices: challenges with individuals with multiple comorbid behavioral and health problems. J Psychol Clin Psychiatry.

[10] Provino R (2010). The role of adaptogens in stress management. Aust J Med Herbalism.

[11] Panossian A, Wikman G (2009). Evidence-based efficacy of adaptogens in fatigue and molecular mechanisms related to their stress-protective activity. Curr Clin Pharmacol.

[12] Chandrasekhar K, Kapoor J, Anishetty S (2012). Safety and efficacy of high-concentration full-spectrum Ashwagandha root extract in reducing stress and anxiety. Indian J Psychol Med.

[13] Dalle-Donne I, Scaloni A, Giustarini D, Cavarra E, Tell G, Lungarella G (2005). Proteins as biomarkers of oxidative/nitrosative stress in diseases. Mass Spectrom Rev.

[14] Ho E, Karimi Galougahi K, Liu CC, Bhindi R, Figtree GA (2013). Biological markers of oxidative stress. Redox Biol.

[15] Raut A, Rege N, Shirolkar S, Pandey S, Tadvi F, Solanki P (2012). Exploratory study on tolerability, safety, and activity of Ashwagandha. J Ayurveda Integr Med.

[16] Tandon N, Yadav SS (2020). Safety and clinical effectiveness of Withania somnifera root. J Ethnopharmacol.

[17] Wang W, Ige OO, Ding Y, He M, Long P, Wang S (2023). Triphala polyphenols and stress resilience. Curr Res Food Sci.

[18] Peterson CT, Denniston K, Chopra D (2017). Therapeutic uses of Triphala. J Altern Complement Med.

[19] Kumar NS, Nair AS, Nair AM, Murali M (2016). Pharmacological and therapeutic effects of Triphala. J Pharmacogn Phytochem.

[20] Dhanalakshmi S, Devi RS, Srikumar R, Manikandan S, Thangaraj R (2007). Protective effect of Triphala on cold stress. Yakugaku Zasshi.

[21] Srikumar R, Parthasarathy NJ, Manikandan S, Narayanan GS, Sheeladevi R (2006). Effect of Triphala on oxidative stress and immunity. Mol Cell Biochem.

[22] Johri RK, Zutshi U (1992). Ayurvedic formulation ‘Trikatu’. J Ethnopharmacol.

[23] Forman-Dolan J, Caggiano C, Anillo I, Kennedy TD (2022). Burnout among corrections professionals. Int J Environ Res Public Health.

[24] Raveendran R (2004). Rando randomization software/manual.

[25] Salve J, Pate S, Debnath K, Langade D (2019). Adaptogenic and anxiolytic effects of Ashwagandha root extract. Cureus.

[26] Cohen S, Kamarck T, Mermelstein R (1983). A global measure of perceived stress. J Health Soc Behav.

[27] Maier W, Buller R, Philipp M, Heuser I (1988). The Hamilton Anxiety Scale: reliability, validity and sensitivity to change in anxiety and depressive disorders. J Affect Disord.

[28] Searight HR, Montone K (2020). Profile of Mood States. Encyclopedia of Personality and Individual Differences.

[29] Hills P, Argyle M (2002). The Oxford Happiness Questionnaire: a compact scale for the measurement of psychological well-being. Pers Individ Dif.

[30] Sengupta P, Agarwal A, Pogrebetskaya M, Roychoudhury S, Durairajanayagam D, Henkel R (2018). Role of Withania somnifera (Ashwagandha) in male infertility. Reprod Biomed Online.

[31] Akhgarjand C, Asoudeh F, Bagheri A, Kalantar Z, Vahabi Z, Shab-bidar S (2022). Ashwagandha supplementation and stress: a systematic review and meta-analysis. Phytother Res.

[32] Li X, Wu L, Wu R, Sun M, Fu K, Kuang T (2022). Ayurveda and Chinese medicinal preparations: therapeutic potential and mechanisms. J Ethnopharmacol.

[33] Baliga MS, Meera S, Mathai B, Rai MP, Pawar V, Palatty PL (2012). Scientific validation of the ethnomedicinal properties of the Ayurvedic drug Triphala: a review. Chin J Integr Med.

[34] Net-Anong S, Phuwajaroanpong A, Plirat W, Konyanee A, Chaniad P, Punsawad C (2025). Triphala, Trikatu, and Benjakul: an evidence-based review of their pharmacology, toxicology, and clinical potential in integrative medicine. J Appl Pharm Sci.

[35] Varma SR, Sivaprakasam TO, Mishra A, Kumar LM, Prakash NS, Prabhu S (2016). Protective Effects of Triphala on Dermal Fibroblasts and Human Keratinocytes. PLoS One.

[36] Phetkate P, Kummalue T, Rinthong PO, Kietinun S, Sriyakul K (2020). Study of the safety of oral Triphala aqueous extract on healthy volunteers. J Integr Med.

[37] Phetkate P, Kummalue T, U-Pratya Y, Kietinun S (2012). Significant increase in cytotoxic T lymphocytes and natural killer cells by triphala: a clinical phase I study. Evid Based Complement Alternat Med.

[38] Chanda D, Shanker K, Pal A, Luqman S, Bawankule DU, Mani D, Darokar MP (2009). Safety evaluation of Trikatu, a generic Ayurvedic medicine in Charles Foster rats. J Toxicol Sci.

[39] Wang W, Ige OO, Ding Y, He M, Long P, Wang S (2023). Triphala polyphenols and stress resilience. Curr Res Food Sci.

[40] Fatima K, Malik J, Muskan F, Raza G, Waseem A, Shahid H (2024). Safety and efficacy of Withania somnifera for anxiety and insomnia. Hum Psychopharmacol.

[41] Kale S, Lopresti A, Suri R, Garg N, Langade D (2024). Ashwagandha for cognition, energy, and mood. J Psychoactive Drugs.

